# Virulence adaption to environment promotes the age-dependent nasal colonization of *Staphylococcus aureus*

**DOI:** 10.1080/22221751.2022.2074316

**Published:** 2022-05-23

**Authors:** Na Zhao, Danhong Cheng, Ziyu Yang, Yao Liu, Yanan Wang, Ying Jian, Hua Wang, Min Li, Taeok Bae, Qian Liu

**Affiliations:** a Department of Laboratory Medicine, Ren Ji Hospital, School of Medicine, Shanghai Jiao Tong University, Shanghai, People’s Republic of China; b Department of Microbiology and Immunology, Indiana University School of Medicine-Northwest, Gary, IN, USA

**Keywords:** *Staphylococcus aureus*, colonization, adaption, virulence, SaeRS

## Abstract

*Staphylococcus aureus* is an important human commensal bacteria colonizing the human body, especially the nasal cavity. The nasal carriage can be a source of *S. aureus* bacteremia. However, the bacterial factors contributing to nasal colonization are not completely understood. By analysing *S. aureus* strains from the nasal cavity of the children, young adults, and seniors, we found that the low activity of the SaeRS two-component system (TCS) is an important determinant for *S. aureus* to colonize in seniors. The senior group isolates of *S. aureus* showed a rather distinct sequence type composition as compared with other age group isolates. The senior group isolates showed not only a lower gene carriage of enterotoxins a, c, and q but also lower hemolytic activity against human red blood cells. Of regulators affecting hemolysin production (i.e. *agr*, *saeRS*, *rot*, *rsp*, and *sarS*), only the SaeRS TCS showed an age-dependent decrease of activity. The decreased virulence and better colonization ability of the senior group isolates of *S. aureus* were confirmed in the mouse model. The senior group isolates showed the lowest survival and the best adhesion and colonizing ability. Also, the senior nasal secretions supported *S. aureus* survival better than the child and young adult nasal secretions. These results indicated that the senior nasal cavity favours colonization of *S. aureus* with higher adhesion and lower virulence, to which the reduced SaeRS TCS activity contributes. Taken together, our results illustrate an example of bacterial adaptation to the changing host environment.

## Introduction

*Staphylococcus aureus* is a Gram-positive pathogen causing various acute and chronic diseases [1]. It is also a commensal bacterium frequently colonizing human anterior nares [2]. Nasal carriage is crucial for endogenous infections and transmission [3]. A series of adhesive molecules contribute to nasal colonization of *S. aureus* [4]. Staphylococcal protein A (SpA) influences the duration of *S. aureus* nasal carriage [5,6]. Clumping factor B determines *S. aureus* nasal carriage by binding to human cytokeratin 10 [7]. The cell wall teichoic acid governs *S. aureus* colonization by interacting with SREC-I receptor on nasal epithelial cells [8]. In contrast, the toxins, including α-toxin (*hla*) and phenol soluble modulin (*psm*), were not transcribed during colonization [9]. The global virulence regulators such as Agr and the SaeRS two-component system (TCS) are not active in the early stage of colonization [9]. Furthermore, the host nasal environment also affects *S. aureus* colonization. For instance, the synthesis of methionine is indispensable for *S. aureus* growth in the nasal environment [10]. The commensal bacteria inhibit *S. aureus* colonization by competing for adhesion sites and nutrients or by producing antimicrobial components [11].

*S. aureus* nasal carriage is a dynamic process [12] and varies with host factors such as age, ethnic groups, and geographical location [13]. In single sampling studies, the nasal carriage rate of *S. aureus* in the adult population varies in different countries from 9.1% to 57.8% [14]. The longitudinal studies typically focus on the different types of *S. aureus* nasal carriers (i.e. persistent, intermittent, and non-carriers) [2]. Children have higher persistent carriage rates than adults [15]. In healthy children, *S. aureus* carriage peaked at 10 years [16]. The evolutionary analysis of *S. aureus* from carriage to infection showed that genetic changes contribute to the transition [17]. For example, a strain with a truncation in Rsp evolved from an asymptomatically nasal colonizer is able to cause bloodstream infection [18]. However, the dynamic interaction between *S. aureus* and the host nasal environment during the bacterial colonization has not been completely understood.

Previously, by culture-based analysis of nasal swabs, we isolated 3 218 Staphylococci from nasal swabs of three groups of human volunteers: 158 children, 210 young adults, and 158 seniors [19]. Among them, ∼85% (2 748 isolates) were coagulase-negative Staphylococci (CoNS), and ∼15% (470 isolates) were *S. aureus*. Intriguingly, we found that *S. aureus* colonization decreases with aging. However, it remains to be determined whether or not the *S. aureus* strains of different age groups have unique traits that optimize their colonization of their corresponding host. Therefore in this study, we systemically analysed and compared the previously isolated *S. aureus* strains and found that the reduced activity of the SaeRS TCS is a primary bacterial trait contributing to the colonization of the senior group.

## Materials and methods

**Ethics statement.** The animal experiment was performed following the Guide for the Care and Use of Laboratory Animals. The red blood cells were collected from the venous blood of healthy individuals. The animal protocol and the human samples collection were approved by the ethics committee of Ren Ji Hospital, Shanghai Jiao Tong University, School of Medicine. The written consent form was received from all human subjects.

**Bacteria.** As reported previously, *Staphylococcus* strains were isolated from the nasal swabs of three groups of healthy volunteers: children (5–6 years), young adults (18–20 years), and seniors (50–90 years) [19]. Among all the *S. aureus* strains, we randomly selected 60 strains from each group (Table S1). Of the 180 *S. aureus* isolates, all ST188 and ST398 strains were used for hemolysis assay and growth curve. For whole-genome analysis, 18 isolates (6 / group) of ST188 were randomly selected. For RT–PCR and animal experiments, 24 isolates (8 / group) of ST188 were randomly selected with a random number generator (Rand function of Perl5). Generally, *S. aureus* isolates were grown in tryptic soy broth (TSB; Oxoid) with shaking at 200 rpm at 37°C.

**Generation of mutant strains.** The *sae*-deletion plasmid pKOR1Δ*sae* was reported in our previous work [20]. The plasmid was electroporated into *S. aureus* RN4220 and subsequently transduced into clinical isolates ST398-N7-25-5. The *sae-*deletion was carried out as described previously [21].

**SpA typing and Multi-Locus Sequence Typing (MLST).** The primers for amplification of *spA* polymorphic X region and seven housekeeping genes were listed in Table S2. The sequences of the PCR products were used for *spA* typing (http://spa.ridom.de/). MLST was performed by detecting the following 7 housekeeping genes: carbamate kinase (*arcC*), shikimate dehydrogenase (*aroE*), glycerolkinase (*glp*), guanylate kinase (*gmk*), phosphate acetyltransferase (*pta*), triosephosphate isomerase (*tpi*), and acetyl coenzyme A acetyltransferase (*yqiL*). Sequences of PCR products were compared with the existing sequences available in the MLST website database (http://www.pubmlst.net). The allelic numbers for PCR sequences were determined by the existing sequences available on the MLST website (http://www.mlst.net).

**DNA extraction.** The bacteria pellets were treated with lysostaphin (50 μg/ml) at 37°C for 30 min. The genomic DNA was extracted by a standard phenol–chloroform extraction protocol. High-quality DNA (OD260/280=1.8∼2.0, > 1 μg) was used for experiments.

**Library construction and sequencing.** DNA samples were sheared into 400–500 bp fragments with Covaris M220 Focused Acoustic Shearer by following the manufacturer’s protocol. Illumina sequencing libraries were prepared from the sheared fragments with the NEXTflex™ Rapid DNA-Seq Kit. First, 5’ ends were repaired and phosphorylated. Next, the 3’ ends were A-tailed and ligated to sequencing adapters. The adapter-ligated products were enriched by PCR. The prepared libraries were then used for paired-end Illumina sequencing (2 × 150 bp) on an Illumina HiSeq X Ten (Majorbio, Shanghai).

Read quality control was conducted with a quality control option in CLC Genomics Workbench 12.0 (QIAGEN, Aarhus, Denmark), and the raw data were filtered before assembly. Sequences containing more than 10% ambiguous N bases or sequences shorter than 30 bp in length were removed. The Illumina sequences of 18 isolates in this study are available in the Sequence Read Archive (BioProject accession number: PRJNA729814).

**SNP calling and phylogenetic construction.** Whole-genome alignment was carried out in CLC Genomics Workbench 12.0 with the default options. Clean reads were obtained after removing the adapter and low-quality sequences. The whole-genome sequence of the *S. aureus* MW2 strain (ST1, Gen-Bank accession code: BA000033.2) was used to map the reads. The maximum likelihood (ML) tree was constructed and annotated with the Interactive Tree Of Life (iTOL) (https://itol.embl.de/).

**Antimicrobial susceptibility testing.** The antibiotic susceptibility of all isolates in this study was performed on Mueller-Hinton agar using the standard disk diffusion method according to the Clinical and Laboratory Standards Institute (CLSI) guidelines [22]. The following antimicrobial agents were tested: penicillin (P), erythromycin (E), tetracycline (TET), clindamycin (DA), gentamicin (CN), oxacillin (OXA), ciprofloxacin (CIP), cefoxitin (FOX), trimethoprim/sulfamethoxazole (SXT), and rifampicin (RF) (Oxoid, UK). *S. aureus* ATCC25239 was used as quality control.

**Biofilm formation assay.** Semi-quantitative biofilm assays were performed as previously described [23]. Briefly, the overnight cultures of *S. aureus* strains were diluted at 1:100 into fresh TSB with 0.5% glucose. The diluted cultures were then pipetted into sterile 96-well flat-bottom tissue culture plates and incubated at 37°C for 24 h. Cells were fixed by using Bouin’s fixative. After 1 h incubation, the fixative was removed, and the wells were washed with phosphate-buffered saline (PBS). Organisms in the wells were then stained with 0.4% (wt/vol) crystal violet, and the floating stain was washed off with slow-running water. After drying, biofilm formation on 96-well plate was measured with a MicroELISA autoreader (BioTek, Synergy 2) at 570 nm.

**Virulence gene profiles.** The presence of the following 35 staphylococcal virulence genes was examined by PCR [24]. All the isolates were tested for the following 35 staphylococcal virulence genes: 10 adhesins: ser-Asp rich fibrinogen-binding protein C (*sdrC*)*,* ser-Asp rich fibrinogen-binding protein E (*sdrE*)*,* clumping factor A (*clfA*)*,* clumping factor B (*clfB*)*,* collagen adhesion (*cna*)*,* intercellular adhesin (*icaA*)*,* elastin binding protein (*ebpS*)*,* fibrinogen binding protein (*efb*)*,* fibronectin-binding protein A (*fnbA*)*,* fibronectin-binding protein B (*fnbB*). 16 staphylococcal enterotoxins (*sea*, *seb*, *sec*, *sed*, *see*, *seg*, *seh*, *sei*, *sej*, *sek*, *sel*, *sem*, *sen*, *seo*, *sep*, *seq*), hemolysins (*hla*, *hlb*, *hld, hlgA, hlgB, hlgC, PSMα*), leukotoxins (*lukE*, *lukS*). The primers are listed in Table S2.

**Hemolysis assay.** The hemolysis assay was conducted as follows [25]. The bacteria were cultured for 8 h in 3 ml TSB with shaking (200 rpm) using 15 ml tubes. The supernatants were collected by centrifuging (14 000 rpm, 10 min). The culture supernatant (100 μl) was incubated with 100 μl human red blood cells (2% [v/v] in PBS) in a 96-well round-bottom plate at 37°C for 1 h. Then, the plate was centrifuged for 10 min at 4 000 rpm. The respective supernatant (100 μl) was transferred into an additional sterile 96-well flat-bottom plate, and OD_540 nm_ was measured with a Synerge 2 autoreader (BioTek).

**Real-time quantitative reverse transcription-PCR.** The *S. aureus* strains were grown to an exponential phase. The cell pellets were broken with a FastPrep-96 (MP Biomedicals Products) at 800 rpm for 300 s three times. After centrifugation, the supernatant was collected for total RNA isolation according to the manufacturer’s instructions (Qiagen). After DNase treatment with a TRUBO DNA-free kit (Ambion), 1 μg of total RNA was reverse transcribed with a Prime Script RT reagent kit (Qiagen). The cDNA was used as a template for RT–PCR with SYBR Green PCR reagents (Roche). Reactions were performed in a MicroAmp optical 96-well reaction plate with a 7500 sequence detector (Applied Biosystems). The primers were listed in Table S2.

**SaePQRS sequencing.** The coding regions of *saeRS* were amplified by primers *saeRS*-F/R. The coding regions of *saePQ* were amplified by primers *saeP*-F/ *saeQ*-R. The PCR products were sequenced using the primers listed in Table S2. The SaePQRS sequence of the *S. aureus* S0385 strain (ST398, Gen-Bank accession code: AM990992.1) was used as a reference.

**Mouse skin abscess model.** The experiment was performed as described [26]. The overnight culture was diluted at 1:100 in fresh TSB and grown at 37°C for 4 h. Bacteria were harvested by centrifugation (6000 rpm, 10 min), washed twice with sterile PBS, and suspended in PBS. Outbred, immune-competent hairless male mice (4∼6 weeks old) were anesthetized with Avertin (Sigma T48402) by intraperitoneal injection and administered with *S. aureus* (10^8^ CFU in 100 μl of PBS) by s.c injection on the back. Each day the skin lesion size was measured by length (L) and width (W). On the 3rd day, mice were killed by anesthesia overdose, and the infected skin tissue was excised and homogenized in 500 μl PBS. The homogenized skin tissue was diluted and plated on 5% sheep blood agar to determine CFU.

**Mouse nasal colonization model.** The experiment was performed as described [19]. BALB/c female mice (4∼6 weeks old) were anesthetized with Avertin; then, the mice were instilled intranasally drop-wise and equally split between the two nostrils. On the 3rd day, the animals were sacrificed, and the noses were surgically removed and homogenized in 500 μl PBS. The homogenized tissues were diluted and then spread on 5% sheep blood agar for CFU determination.

**Adhesion and invasion assay.** *S. aureus* was grown to the exponential growth phase in TSB and washed twice with Dulbecco's Modified Eagle Medium (DMEM). The human nasal epithelial cell (RPMI2650) was cultured at 37°C in DMEM supplemented with fetal bovine serum (FBS, 10%) and 5% CO_2_. The cells were infected with *S. aureus* at a multiplicity-of-infection (MOI) = 50.

For the adhesion/invasion assay, the cells were incubated for 2 h; then, the cells were collected, washed twice with PBS, and lysed with the addition of 0.1% deoxysodium cholate solution (500 µl). The bacterial colony-forming unit (CFU) was enumerated by serial dilution of the epithelial cell lysates and spreading of the diluted lysates on 5% sheep blood agar.

The invasion assay was carried out as described by Kim et al. [27]. Briefly, after incubation with the bacteria for 2 h, the cells were washed with PBS twice and then incubated in DMEM medium supplemented with lysostaphin (8.8 nM) for 2 h. Then the lysostaphin was quenched by 50 mM EDTA (pH 8.7). The cells were washed twice with PBS and lysed with the addition of 0.1% deoxysodium cholate solution (500 µl). Bacterial CFU was enumerated by serial dilutions of epithelial cell lysates and spreading of the lysates on 5% sheep blood agar.

***In vitro* killing assay.** Three groups of healthy volunteers were recruited from the community of Shanghai, China. In total, 8 children (5–7 years old), 10 young adults (18–20 years old), and 10 seniors (70–90 years old) were enrolled. Nostril swabs were obtained, submerged in 1 ml sterile saline, and vortexed for 2 min. The resulting nostril swab suspensions (100 μl) were spread on 5% sheep blood agar to identify *S. aureus* carriers. Two children and two seniors were found to carry *S. aureus*, and those samples were excluded from the killing assay described below.

For bacterial subjects, we used the following four *S. aureus* strains collected in our previous study [19]: ST398-N7-25-5 from the child group and its *sae* mutant, ST398-N118-1 from the young adult group, and ST398-NO105-9 from the senior group (Table S1). Overnight cultures of the test strains were diluted 1:100 in fresh TSB and grown at 37°C for 2 h. Bacteria cells were harvested by centrifugation (6000 rpm, 10 min), washed twice with sterile saline, and suspended in the same solution. The bacterial cells (∼10^4^, 2 μl) were mixed with the nostril swab suspension (200 μl), and the resulting mixtures were incubated at 37°C, collected at designated time points, diluted, and spread on 5% sheep blood agar for CFU determination. For blank control, the synthetic nasal medium (SNM3) was used [10].

**Statistical Analysis.** For the comparison between the two groups, the statistical analysis was performed by unpaired, two-tailed Student’s t-test (Prism version 7.0). One-way ANOVA and post hoc tests (Tukey's tests) were used for three-set data analysis. Error bars indicated the standard deviation (±SD), and *P*-values < 0.050 were considered statistically significant. *P < 0.05, **P < 0.01, ***P < 0.001, ****P < 0.0001.

## Results

### The characteristics of senior group isolates of S. aureus are rather distinct

**Figure 1.**
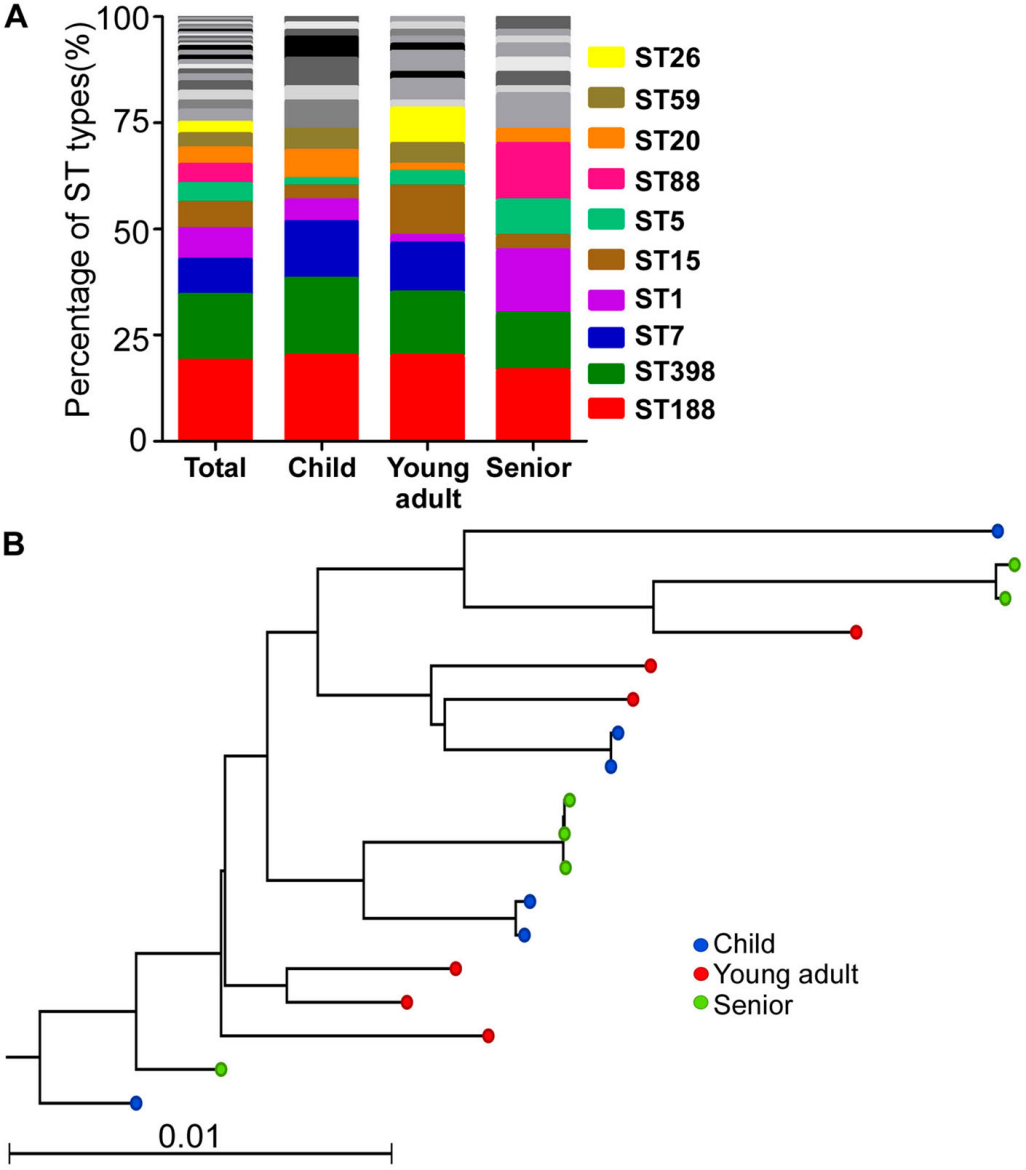
The molecular epidemiological characteristics of *S. aureus* strains isolated from nares. (A) The MLST types of the test strains. (B) Maximum likelihood tree of ST188 clonotype *S. aureus* isolates. This tree was built with a maximum likelihood method with a GTR substitution model utilizing SNPs of the 18 isolates. The NGS raw data were mapped with the genome of *S. aureus* reference MW2.

Previously, we compared the bacteria composition in nares of different groups (i.e. children, young adults, and seniors), which are representative of ages with not-yet established, mature, and declining immune systems, and found that *S. aureus* colonization decreases with aging [19]. To identify the underlying mechanism of the age-dependent *S. aureus* colonization, we examined the molecular epidemiological characteristics of the colonized strains. In total, we collected 470 isolates: 264 strains from children, 113 from young adults, and 93 from seniors. Here, all *S. aureus* isolates for each group were given a number, and 60 isolates were randomly selected from each group and subjected to MLST to determine the sequence types (ST) of the *S. aureus* isolates. A total of 32 STs were identified. Overall, the top three STs were ST188 (18.9%, 34/180), ST398 (15.6%, 28/180), and ST7 (8.3%, 15/180). ST188 was predominant in all groups. ST398 was the 2nd dominant type in the child and young adult group, whereas ST1 was the 2nd dominant type in the senior group (Figure 1(A)). Interestingly, although ST7 was the 3^rd^ most dominant sequence type in the whole population, no ST7 type was found in the senior group. The *spA* typing showed that most of the ST188 strains were t189 (73.5%, 25/34). All ST398 strains isolated from the senior group were t034, whereas the *spA* types of the ST398 strains isolated from the child and the young adult group varied (Table S1).

### The lineage of ST188 strains does not show any age-dependent pattern

To examine whether each age group was colonized by the unique lineage of ST, we randomly selected six isolates of ST188 from each age group and sequenced their whole genome (Table S1). In total, 17 638 core genome SNPs were identified. The maximum likelihood tree analysis, however, showed no distinct clades among the three groups (Figure 1(B)).

### Antibiotic resistance, biofilm formation capability, and growth rates are not significantly different among the age-group isolates

Next, we examined the antibiotic resistance of the 180 *S. aureus* isolates of the three groups to ten commonly used antibiotics. The overall antibiotic resistance varied among the age group isolates, and no age-dependent pattern was observed (Figure S1A).

Since biofilm formation is critical for successful nasal colonization of *S. aureus* [28], we compared the biofilm formation capability of the isolated strains by semi-quantitative biofilm assay [23]. As shown, the biofilm formation capability was not significantly different among the three age-group isolates (Figure S1B).

We also compared the growth of two predominant ST types (i.e. ST188 and ST398) of the isolates. In TSB, no significant difference was observed (Figure S1C). However, although not statistically significant, the senior group isolates showed a trend of higher growth rate in a synthetic nasal medium (SNM3) [10] (Figure S1D).

### The hemolytic activity of the isolates showed an age-dependent decrease

The bacterial virulence factors play an important role in defense against host immune attacks. Therefore, we examined the gene carriage rates of 35 virulence factors in all 180 isolates. Adhesins are one of the main components affecting *S. aureus* initial colonization [29], so we first analysed the gene carriage rates of adhesins. For all the tested 10 adhesins, *cna* showed a significantly lower carriage in the senior group isolates; however, its carriage rate was not different between the child and young adult groups (Figure S2 and Table S3). The carriage rates of all nine remaining adhesins were not different among the age groups.

**Figure 2.**
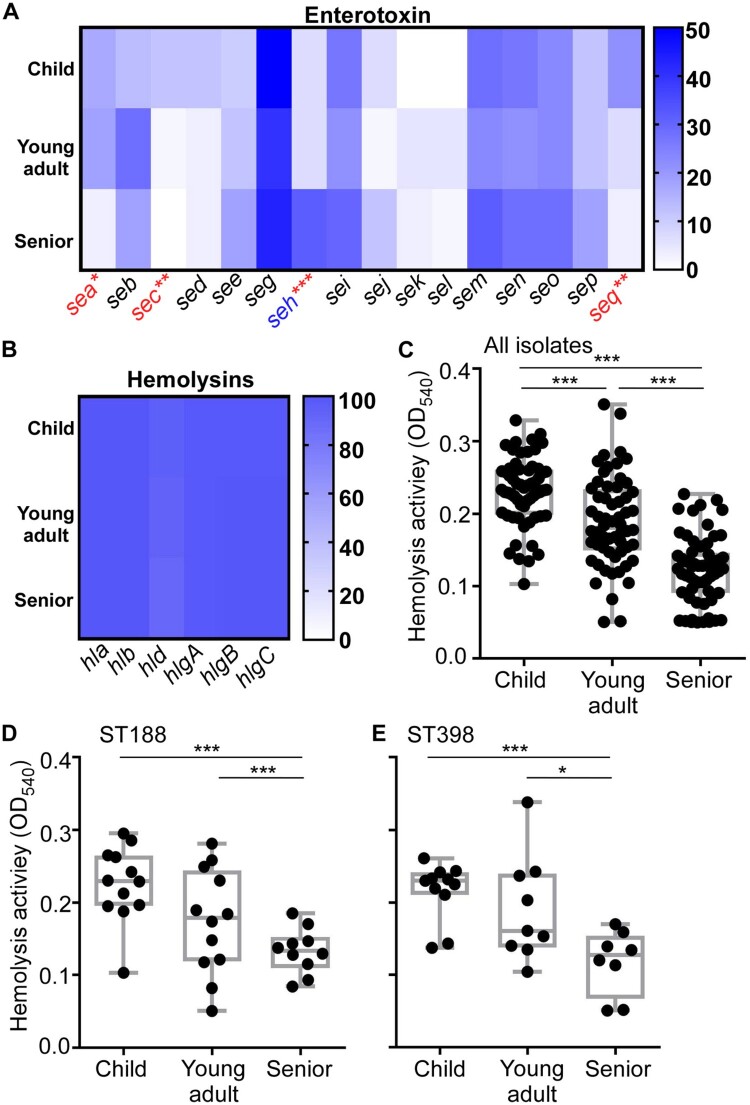
The content of virulence genes (A-C) and hemolytic activity of *S. aureus* isolates (D-F). (A-C) The gene content was determined by PCR for 16 enterotoxins 10 adhesins (A), and 6 hemolysins (B). The red-colored genes show a significantly lower occurrence in the senior isolates, whereas the blue-colored gene shows a higher occurrence. (C-E) The hemolytic activity of all *S. aureus* isolates (C), ST188 (D), and ST398 (E). The results are representative of three independent experiments with four replicates. The statistical significance was measured by the one-way analysis of variance (ANOVA). Error bars show the mean ± SD (*p < 0.05; **p < 0.01; ***p < 0.001).

Of the 16 enterotoxin genes (a-e, g-q) tested, *sea*, *sec*, *seh*, and *seq* showed significantly distinct frequency among the age groups. Intriguingly, of the four, the frequency of *seh* was the highest in the senior group, whereas the other three were the lowest (Figure 2(A) and Table S3). For leukocidins, over 80% strains carried *lukE* gene, while around 10% strains carried *lukS* gene (Table S3). All the strains carried PSMα, and over 90% strains carried the genes with hemolytic activity (Figure 2(B) and Table S3). Nonetheless, none of those leukocidin and *psm* genes showed distinct frequency among the age groups.

Although we observed different carriage rates for several virulence factors in different age group isolates, they do not explain the age-dependent decrease of *S. aureus* nasal colonization. We hypothesized that the age-dependent colonization of *S. aureus* is determined not by the carriage but by the expression of the virulence genes. Since hemolysins are commonly regulated by the global virulence regulators in *S. aureus* [30], we measured the hemolytic activity of the age-group isolates on human red blood cells as an indicator of the virulence gene expression. Intriguingly, the hemolytic activity of *S. aureus* isolates showed an age-dependent decrease (Figure 2(C)), regardless of ST (Figure 2(D,E)), suggesting that the expression of virulence genes, including hemolysins, might contribute to the age-dependent colonization of *S. aureus*.

### Sae contributes to the age-dependent pattern of the hemolytic activity of S. aureus

**Figure 3.**
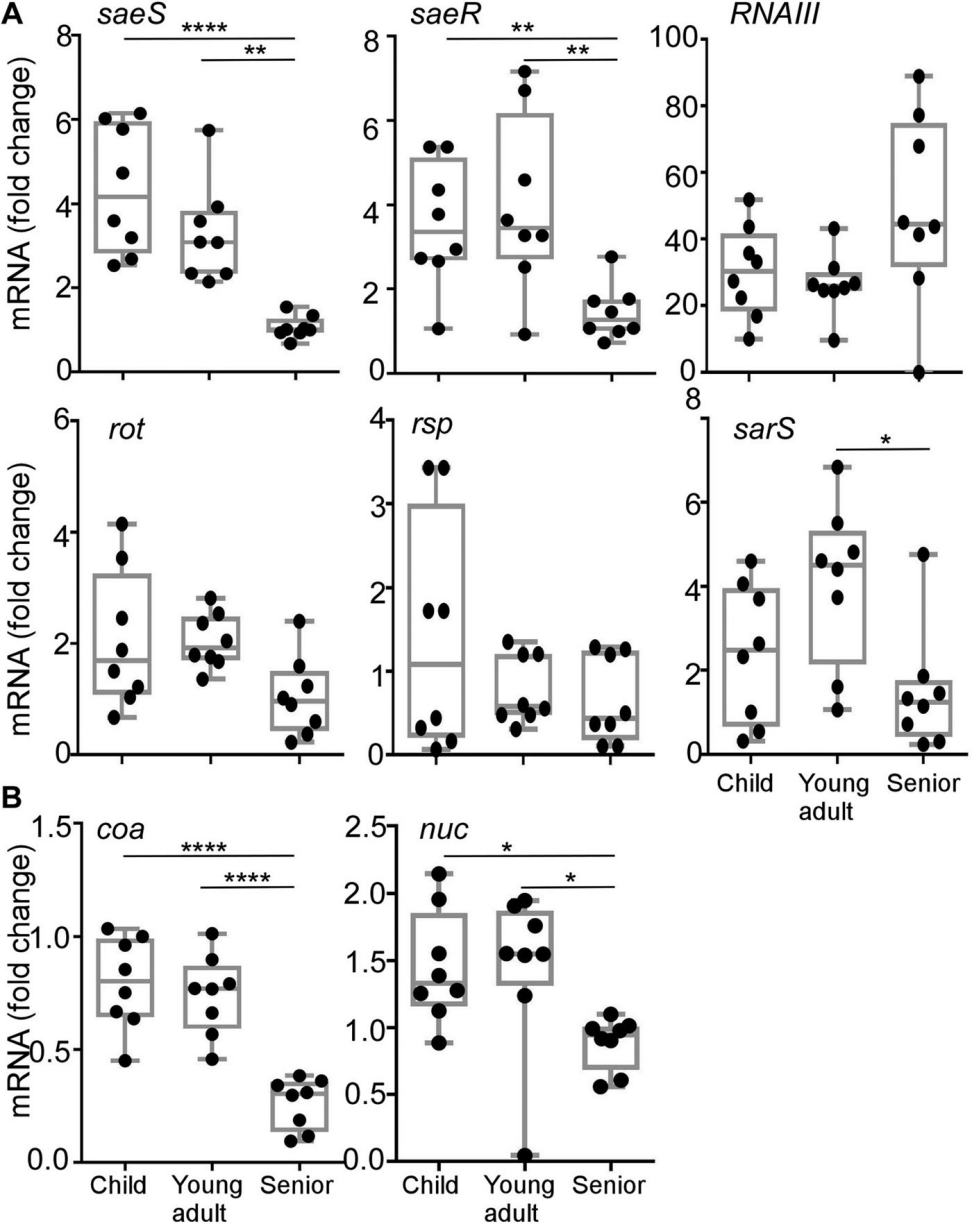
The transcription profiles of hemolysin regulators (A) and the Sae target genes (B). Cells were grown in TSB to exponential growth phase (OD_600 _= 2); then, the transcript levels were determined by qRT-PCR for *saeS, saeR, RNAIII, rot, rsp, sarS, coa,* and *nuc* genes. The *coa* and *nuc* genes are known Sae-targets. The statistical significance was measured by the one-way analysis of variance (ANOVA) (*p < 0.05; **p < 0.01; ****p < 0.0001).

In *S. aureus*, the expression of hemolysins and leukocidins is coordinately controlled by multiple regulators such as *agr*, *saeRS*, *rot*, *rsp,* and *sarS* [30–32]. Therefore, we further examined the transcription levels of those regulators. Eight strains of ST188 were randomly selected from each age group isolates, and the transcript levels of the five regulators were measured. Intriguingly, the transcription of both *saeS* and *saeR,* encoding the sensor kinase and response regulator of the SaeRS TCS, displayed an age group-dependent decrease and was lowest in the senior group isolates (Figure 3(A)). In contrast, no such pattern was observed for the other four regulators (Figure 3(A)). In addition, the age-dependent decreasing of *saeS* and *saeR* transcription was also confirmed in all ST398 strains from each group isolates (Figure S3). To further verify that the decreased *saeS* and *saeR* transcription represents the lower SaeRS TCS activity, we measured the transcript levels of two well-studied Sae-target genes: coagulase (*coa*) and nuclease (*nuc*) [33,34]. As with s*aeS*, both Sae-target genes showed a lower transcript level in the senior group (Figure 3(B)). These results indicate that the senior group isolates have lower Sae activity.

### Genomic mutations are not responsible for the lower Sae-activity of the senior group isolates

To examine whether the lower Sae activity is due to mutations in the genome, we analysed the DNA sequence of the *saePQRS* region of all the 34 strains of ST188 and 28 strains of ST398 used in the hemolytic assay. Indeed, we observed several SNPs for both SaeP and SaeQ. For *saeP* gene, 4 SNPs (t50c, t159a, a181 g, t273c) were observed in 32 strains of ST188, while one common SNP (a181 g) was found in all ST398 strains. We also observed *saeP* t50c and *saeP* g114a/t273c from strains isolated from child and young adult groups, respectively. For *saeQ* gene, 5 SNPs (c109t, a123c, a205 g, t210c, t216c) were observed in all ST188 strains and 1 ST398 strain from the young adult group (Table S1). The SNPs of *saeP* t50c/a181 g and *saeQ* a205 g caused the point mutants for the proteins: SaeP I17 T/S61G, and SaeQ I69 V, however, there are no general alterations for SaePQ sequences in the senior group.

Only one ST188 strain from the young adult group carried an SNP (c95a) in the *saeR* gene, resulting in a mutant SaeR protein (i.e. SaeR T32N). Although several SNPs were also identified in the *saeS* gene (t66c, g669a, a885t, t996c), none of them affected the amino acid sequence of the protein. Taken together, our data suggested that the lower Sae-activity in the senior group isolates is not likely due to genomic mutations.

### The senior group isolates display lower survival during host infection

**Figure 4.**
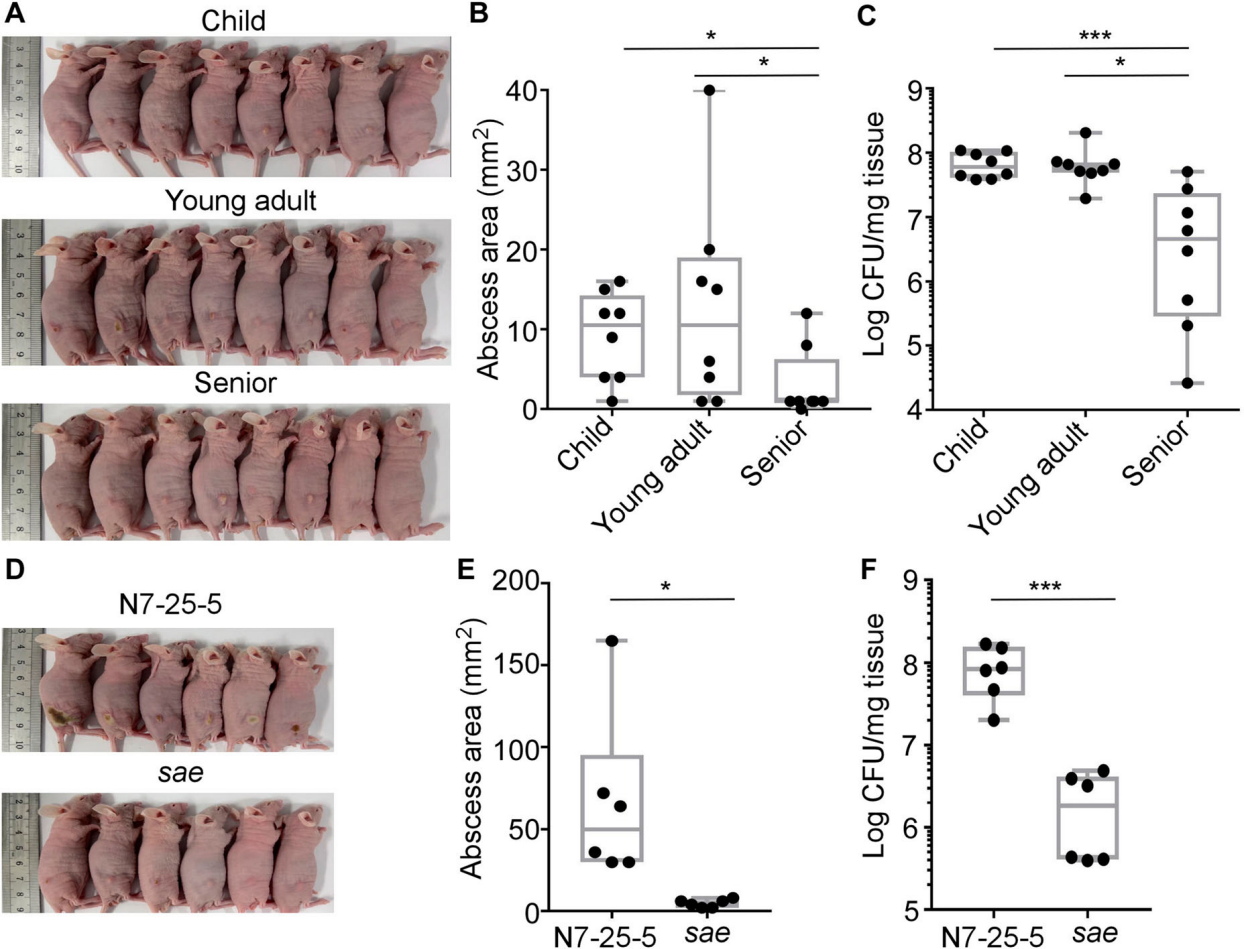
Comparison of the virulence of the *S. aureus* isolates in a murine skin abscess model. (A) The infected mice on day 3 post-infection. Eight strains were randomly selected from the isolates of each group and subjected to the virulence test. Each mouse (one mouse/isolate) was intradermally injected with the test strain (10^8^ CFU) on the left dorsum. (B) The abscess size (i.e. length (L) × width (W)) on day 3 post-infection. (C) CFU normalized by the weight of tissues in the lesion on day 3 post-infection. (D) Effect of *sae-* deletion on bacterial virulence test. ST398-N7-25-5 and its *sae*-deletion mutant (10^8^ CFU) were administered intradermally on the left dorsum of six mice. The infected mice (6 mice for N7-25-5 and 6 mice for the *sae* deletion mutant) were shown at day 3 post-infection. (E) The abscess size (i.e. length (L) × width (W)) on day 3 post-infection. (F) CFU normalized by the weight of tissues in the lesion on day 3 post-infection. The statistical significance was measured by the one-way analysis of variance (ANOVA) or unpaired t-test (*p < 0.05; **p < 0.01; ****p < 0.0001). Error bars show the mean ± SD.

The Sae activity is essential for *S. aureus* virulence [31]. To examine whether the lower hemolytic and the Sae activity in the senior group isolates are conveyed into lower virulence, we subjected the 8 strains of ST188 from each group used for RT–PCR analysis to a murine skin abscess model. On day 3 post-infection, the average size of abscesses caused by the child and young adult group isolates was 9.81 ± 6.50 mm^2^ and 10.13 ± 9.24 mm^2^, respectively, while the average size was 3.38 ± 4.10 mm^2^ for the abscesses caused by the senior group isolates (Figure 4(A,B)); Moreover, the senior group isolates showed significantly lower survival than the child and young adult group isolates (Figure 4(C)), indicating that low virulence strains of *S. aureus* are enriched in seniors nares.

The role of Sae during host infection was further tested. Despite our multiple trials, we could not generate a *sae-*deletion mutant of ST188, the predominant colonizer. Therefore, we generated a *sae-*deletion mutant for a strain of the second dominant sequence type (i.e. ST398-N7-25-5). In a murine skin abscess model, the *sae-*deletion mutant strain displayed significantly decreased abscess (Figure 4(D,E)) and bacterial loads (Figure 4(F)), confirming the crucial role of Sae in bacterial virulence.

### The lower Sae activity correlates with higher colonization efficiency

**Figure 5.**
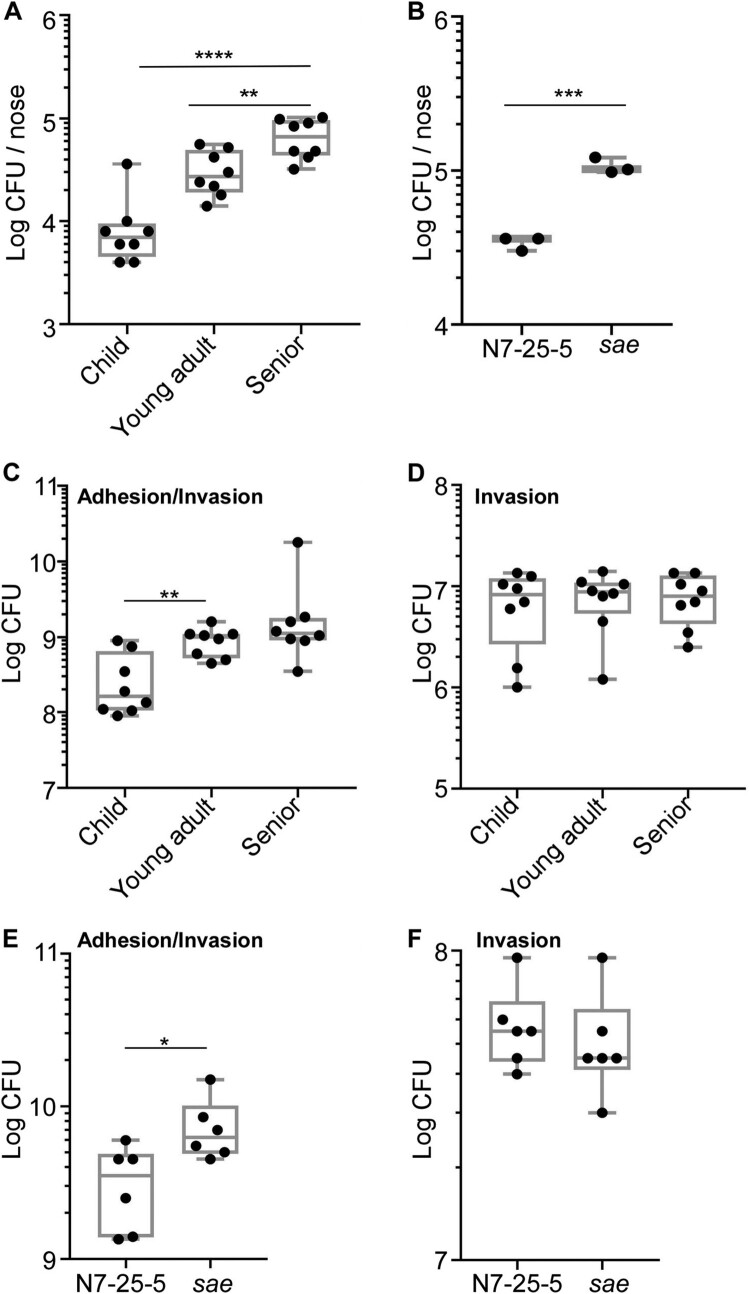
Comparison of the colonization and adhesion of the *S. aureus* isolates. (A) The senior group isolates better colonize mouse nares. Eight strains were randomly selected from the isolates of each group and subjected to the mouse colonization model. The test strains were grown in TSB until the exponential growth phase. After washing, the bacteria (10^8^ CFU) were administered into the nares of each mouse (one mouse/isolate).The colonized bacteria in the nares were determined on day 3 post-infection. The statistical significance was measured by the one-way analysis of variance (ANOVA). **p < 0.01; ****p < 0.0001. (B) The effect of the *sae-*deletion on the nasal colonization of *S. aureus.* The test strain ST398 (N7-25-5) and its *sae*-deletion mutant (*sae*) (10^8^ CFU) were administered into the nares of the mice (*n* = 3). Statistical analysis was carried out by unpaired t-test (***p < 0.001). Error bars show the mean ± SD. (C) The isolates from the senior group displayed better adhesion to nasal epithelial cells. The epithelial cells were incubated with the bacterial strains for 2 h; then, the CFU of bacteria associated with RPMI2650 epithelial cells was measured. (D) The invasion of bacteria does not affect the age group-dependent CFU increase shown in (C). In the invasion assay, external bacteria were eliminated by lysostaphin treatment before CFU counting. The statistical significance was measured by the one-way analysis of variance (ANOVA). **p < 0.01. (E) Sae negatively affects staphylococcal adhesion to RPMI2650 cells. (F) Sae does not affect staphylococcal invasion into RPMI 2650. The data are representative of two independent experiments. Statistical analysis was carried out by unpaired t-test (*p < 0.05).

Next, we examined the nasal colonization ability of 8 strains of ST188 using a mouse model. An equal number of the test strains (10^7^ CFU in 20 μl of PBS) were inoculated into both nostrils of the mice; then, at 3 days of post-inoculation, animals were sacrificed, and the CFU of the nose was measured. Intriguingly, the test strains showed a host age-dependent increase of CFU (Figure 5(A)). The CFU of the young adult group isolates was significantly higher than that of the child group isolates. Furthermore, the senior group isolates displayed the highest CFU than other group isolates (Figure 5(A)). When the mouse experiment was repeated with the WT and the *sae* mutant strain of ST398, the *sae-*deletion mutant displayed significantly higher CFU recovery (Figure 5(B)), indicating that the lower Sae activity of the senior group isolates enhances their colonization efficiency.

The first step of colonization is to adhere to host tissue. To investigate whether the *S. aureus* isolates show the host-dependent increase of adhesion capability, we carried out a cell adhesion/invasion assay. The eight strains of ST188 from each group were incubated with human nasal epithelial cell line RPMI2650 for 2 h, washed twice, and the bacterial cells associated with the host cells-both inside and outside-were enumerated. Indeed, a trend of age-dependent increase in CFU was observed (Figure 5(C)). The young adult isolates showed significantly higher CFU than the child group isolates. Although the senior group isolates showed a higher CFU average count than the young adult group isolates, it was not statistically significant. When the CFU counting was carried out after eliminating bacteria adhering to the outside of host cells with lysostaphin treatment, no significant difference was observed in CFU counts of the *S. aureus* isolates (Figure 5(D)), suggesting that the trend of host age-dependent increase of CFU in Figure 5(C) is due to the increased adherence of the senior group isolates to the host cells. When the experiment was repeated with the WT and the *sae* mutant of ST398, a similar pattern was observed. The *sae* mutant showed a higher association with the host cells without affecting invasion (Figure 5(E,F)), further supporting the idea that the reduced Sae activity in senior group isolates enhances the bacterial colonization via increased adhesion capability.

### The senior nasal secretion provides a better environment for S. aureus survival

**Figure 6.**
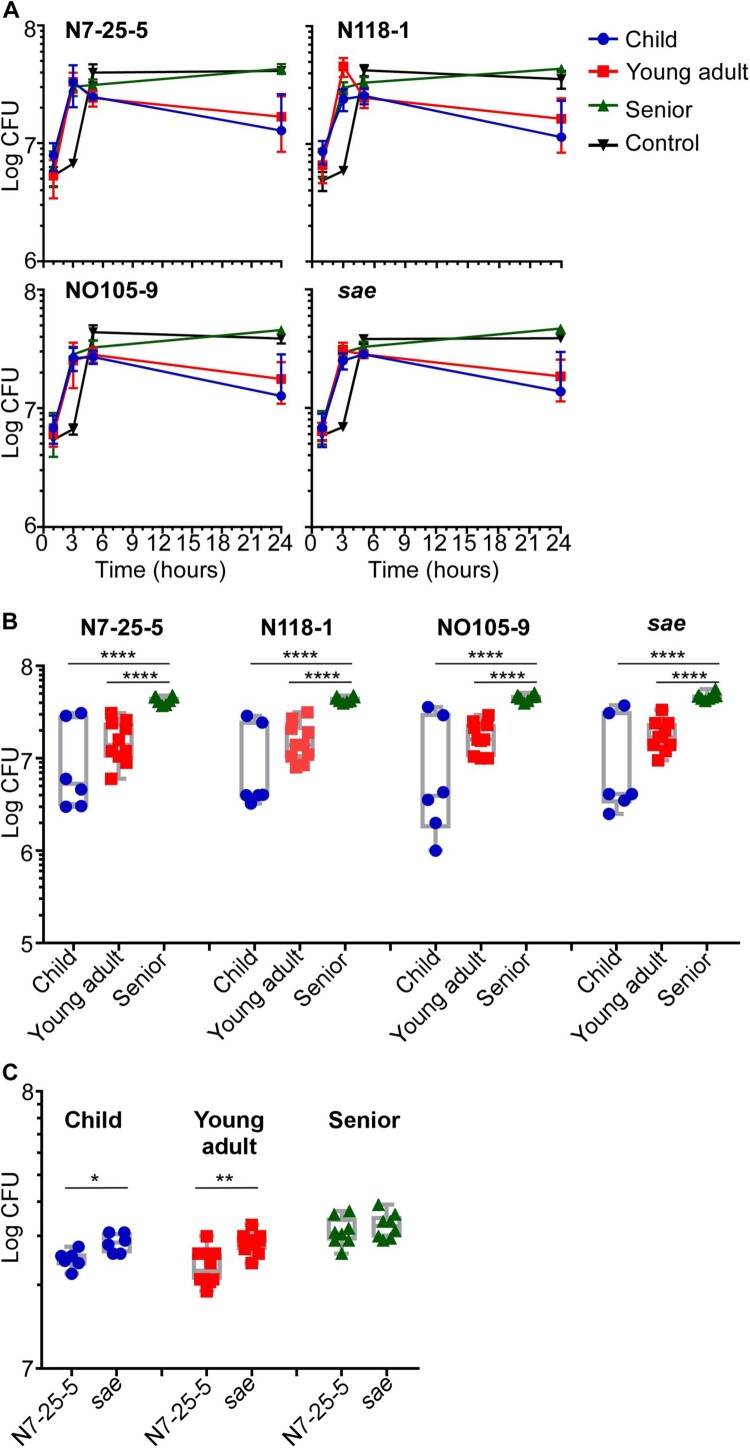
Survival of *S. aureus* in nasal secretion suspensions. Nasal secretions were collected from children, young adults and seniors and suspended in PBS. Four *S. aureus* strains were used for the assay: ST398-N7-25-5 and its *sae* mutant, ST398-N118-1, and ST398-NO105-9. The test strains were incubated in the nasal secretion suspensions, and their survival was measured by CFU counting. (A) Average CFU counting; (B) CFU counting of individual samples in the 24 h samples; (C) CFU counting of ST398-N7-25-5 and *sae-*deletion mutant in nasal secretion samples from children, young adults, and seniors after incubation for 5 h. Control, the synthetic nasal medium (SNM3) was used as blank control. The statistical significance of the survival difference was examined by an unpaired t-test. *p < 0.05; **p < 0.01; ****p < 0.0001. The error bars represent the mean ± SD.

The enrichment of lower virulence strains in the senior group prompted us to compare the antimicrobial activity of nasal secretion from different age groups. If the antimicrobial activity of nasal secretion is reduced with aging, it might allow the colonization of low virulence strains. We used the following four *S. aureus* strains to examine this hypothesis: ST398-N7-25-5 from the child group and its corresponding *sae-*deletion mutant strain, ST398-N118-1 from the young adult group, and ST398-NO105-9 from the senior group. We incubated the four test strains in the nasal secretion suspension from children, adults, and seniors, respectively, and determined CFU at designated time points. Unexpectedly, all the nasal secretion suspensions did not show significant antimicrobial activity, and *S. aureus* grew until 5 h. Intriguingly, however, at 24 h post-incubation, a significantly higher number of *S. aureus* cells survived in nasal secretion suspension of seniors, regardless of strains (Figure 6(A,B)). Also, the final bacterial numbers in the senior nasal secretion were similar to those in the synthetic growth medium (Control vs. Senior in Figure 6(A)). These results indicate that the nasal secretion of seniors provides a better environment for staphylococcal survival than children and young adults’ nasal secretion.

We further investigated the role of Sae in bacterial survival in nasal secretion. ST398-N7-25-5 and its *sae*-deletion mutant were incubated with nasal secretion from three age groups. The *sae*-deletion mutant appears to grow faster than the WT strain in children and young adults nasal secretions, resulting in and showing significantly higher CFU counts by 5 h incubation (Figure 6(C)). However, in senior nasal secretions, both the WT and the *sae*-mutant strains showed a similar growth rate (Figure 6(C)). Also, when the CFU count was measured at 24 h post-incubation, no significant difference was observed between the samples, indicating that growth rate alone cannot explain the enrichment of *S. aureus* strains with reduced Sae activity in the senior group.

## Discussion

*S. aureus* nasal colonization is a risk factor for future infections [35,36]. Most studies have focused on *S. aureus* carriage in a specific population such as infants, children, medical students or patients [15,16,37–39]. However, the comparison of *S. aureus* nasal colonizers isolated from different age groups has rarely been reported. In this study, by examining the characteristics of *S. aureus* strains colonizing different-age populations, we found that lower virulence strains are enriched in the senior population. The most interesting aspect is that the reduced activity of the Sae was associated with the successful colonization of *S. aureus* in seniors’ nares. Although the age range in the senior group was extensive (i.e. 50–90 years), we can define this age group as a representation of people with declining immune status. Our data in the mouse model further corroborated the idea that Sae activity is one of the crucial determinants for the age-dependent nasal colonization of *S. aureus*.

The carriage rate of *S. aureus* appears to decrease with aging, which is likely due to losing the competition with other dominant microbes such as *S. epidermidis* [19]. Intriguingly, the isolates from the senior population displayed a rather distinct ST composition (Figure 1(A)). These might indicate that the senior nasal environment is different from that of other age populations. In fact, the nasal secretion of the senior population seems to support staphylococcal survival better than that of young adults (Figure 6). Therefore, the enrichment of lower virulence strains in senior nares might be the result of bacterial adaptation to the environment.

*S. aureus* nasal colonization is a multifactorial event [29]. Both the local inflammatory response and nasal microenvironment contribute to *S. aureus* colonization [40,41]. For example, the secretion of antimicrobial molecules contributes to *S. aureus* clearance [42], while host factors such as hemoglobin promote nasal colonization [43]. The main component of nasal microbiota is staphylococci [19]. The antimicrobial activity of nasal staphylococci is often high [44]. The nasal *S. lugdunensis* produces the antibiotic lugdunin to reduce *S. aureus* carriage rate [45]. However, in our study, the nasal secretions of children, young adults, and seniors did not show any significant antimicrobial activity under the condition we utilized (Figure 6(A)). It might be possible that the dilution of nasal secretions reduced the antimicrobial activity. However, the better survival of *S. aureus* in the senior nasal secretions indicates that the senior noses provide less harsh conditions for colonizing *S. aureus* (Figure 6(B)), which might explain the enrichment of *S. aureus* strains with lower virulence. It remains to be determined what nasal components are responsible for the better survival of *S. aureus*.

In our study, the dominant genotype of *S. aureus* colonizing nares was ST188. ST188 lineage displayed strong adhesion and biofilm ability [46]. ST398 was the 2nd dominant genotype. ST398 lineage is known to display higher virulence which can cause fatal infection [47]. The successful colonization of ST398 indicated that not only the adhesion ability or biofilm formation but also the virulence contribute to bacteria carriage. ST7, which is the 3rd dominant genotype in children and young adults, was not isolated from seniors. Although it remained to be determined, the senior nasal cavity might provide a harsh condition for the colonization of ST7 strains.

The production of hemolysin is affected by multiple virulence regulators [30]. The naturally occurring polymorphisms in Rsp regulator are associated with colonization and the following infection [48]. Agr dysfunction is prevalent among colonizing *S. aureus* strains [49]. PSMα, which is regulated by Agr, has cytolytic properties for human neutrophils and human erythrocytes [50]. We did not observe any differences either in the carriage of *PSMα* gene or in the transcription of *RNAIII* among the three groups (Figure 3(A)), indicating that Agr system is not involved in the age-dependent decreasing hemolysis. However, in this study, we found that only the Sae activity correlates with the hemolytic activity. Sae plays a key role in *S. aureus* pathogenesis by activating the production of numerous virulence factors such as Coa, Nuc, and Hla [33,51]. The histidine kinase SaeS shows polymorphisms in different clinical isolates, such as SaeS^P^ (L18P) in the Newman strain, SaeS^SK^ (N227S and E268 K), and SaeS^SKT^ (N227S, E268 K, and S351 T), which affects Sae activity [31]. However, SaeS polymorphism cannot explain the reduced Sae activity of the senior isolates. The exact mechanism of the altered Sae activity remains to be elucidated. Nonetheless, the increased nasal colonization by the *sae-*deletion mutant (Figures 5 and 6) strongly suggests that Sae plays a role in nasal colonization.

Finally, based on the experimental results, we hypothesize that in senior nares, *S. aureus* is under increased competition from other bacteria, particularly from *S. epidermidis*, possibly due to the favourable condition for bacterial growth (Figure 6). In such an environment, *S. aureus* strains with reduced Sae activity are preferentially selected because of their increased adhesion to human nasal epithelial cells (Figure 5), resulting in enrichment in senior nares.

## Supplementary Material

Supplemental Material
