# Supplementary material for: Virulence adaption to environment promotes the age-dependent nasal colonization of Staphylococcus aureus
Source: Emerg Microbes Infect. 2022 May 23;11(1):1402–15. doi: 10.1080/22221751.2022.2074316 (PMC9132443; doi:10.1080/22221751.2022.2074316)
Supplement: Supplemental Material [file TEMI_A_2074316_SM9917.zip › Supplemental/Supplemental Material FOR Publication _1.docx]

**Supplementary Figure 1. The general characteristics of *S. aureus* colonized in human nares. (A)** The antibiotic resistance of strains isolated from different age groups. P: penicillin; OXA: oxacillin; FOX: cefoxitin; TET: tetracycline; E: erythromycin; DA: clindamycin; CN: gentamicin; CIP: ciprofloxacin; SXT: sulfamethoxazole; RF: rifampicin. **(B)** Biofilm formation by *S. aureus* colonizing in human nares. Biofilm formation of was evaluated by a semi-quantitative biofilm assay using absorbance at 570 nm *in vitro*. The growth curve of *S. aureus* isolates from different age groups. All ST188 and ST398 strains (Table S1) were used for the growth curve. The test strains were grown in TSB **(C)** or SNM3 **(D)**, and OD_600_ was measured for 8 h or 24 h. The plot (mean + SD) combines the OD_600_ for all isolated at each time point.

**Supplementary Figure 2. The content of adhesions of *S. aureus* isolates.** The gene content was determined by PCR for 10 adhesins. The red-colored genes show a significantly lower occurrence in the senior isolates. The statistical significance was measured by the one-way analysis of variance (ANOVA). Error bars show the mean ± SD (*, p<0.05).

**Supplementary Figure 3. The transcription profiles of *saeS* for ST398 lineage.** Cells were grown in TSB to exponential growth phase (OD_600_ = 2); then , the transcript levels were determined by qRT-PCR for *saeS*. The statistical significance was measured by the one-way analysis of variance (ANOVA) (*, p <0.05; ****, p <0.0001).

**Figure S1**

**
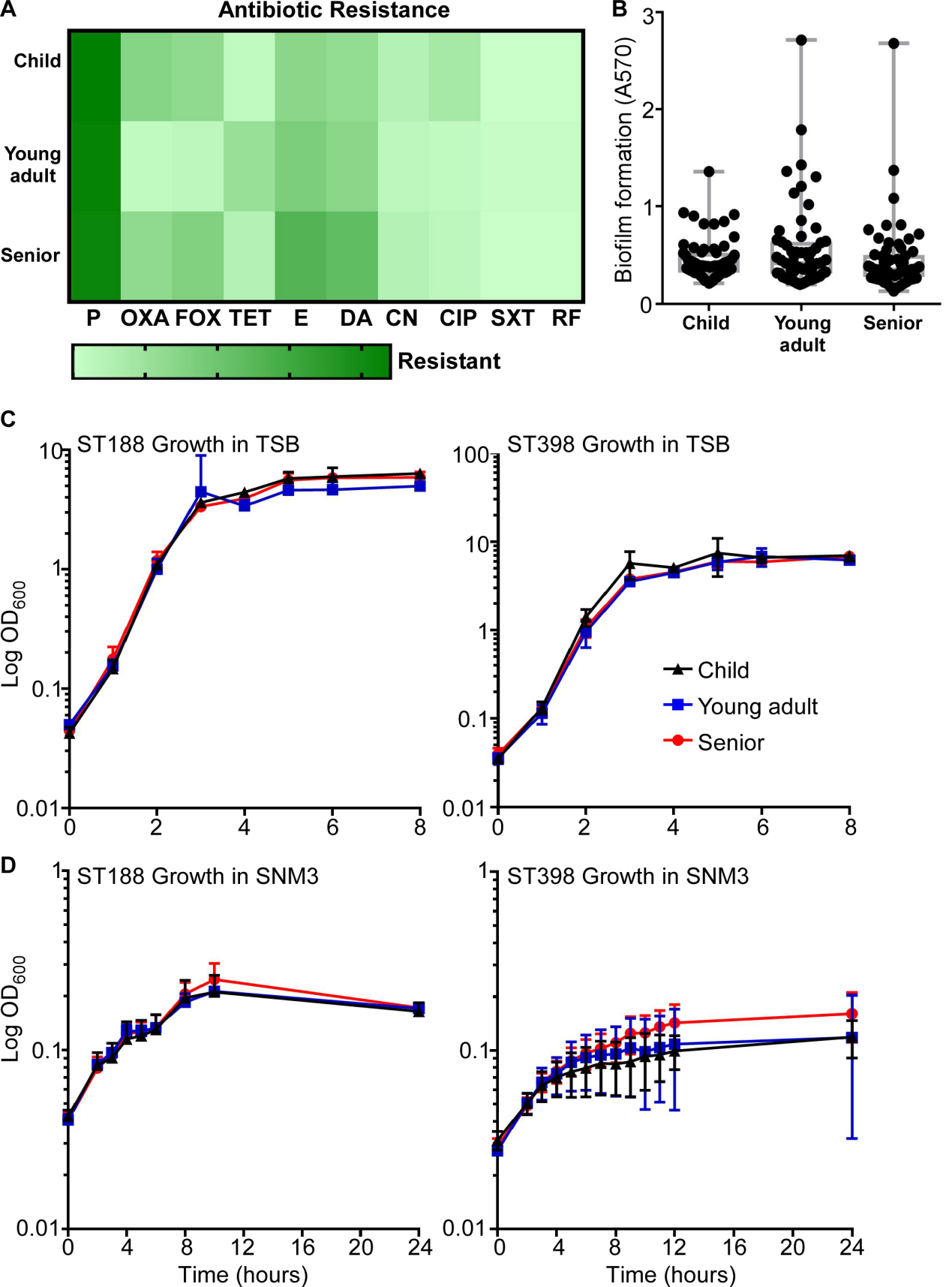
**

**Figure S2**

**
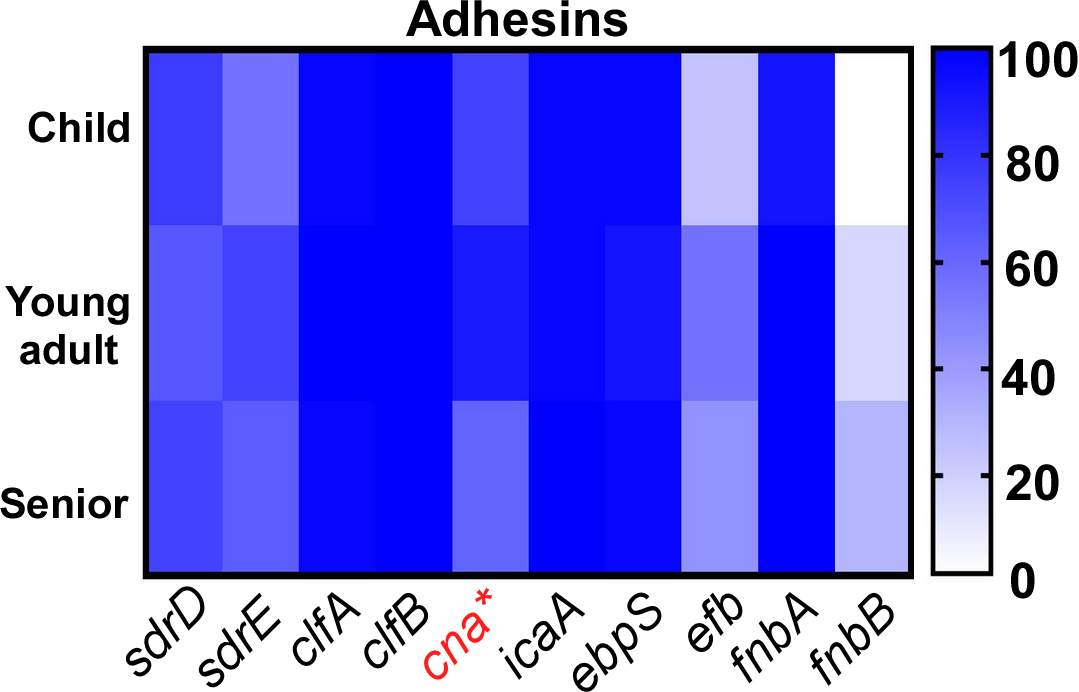
**

**Figure S3**

**
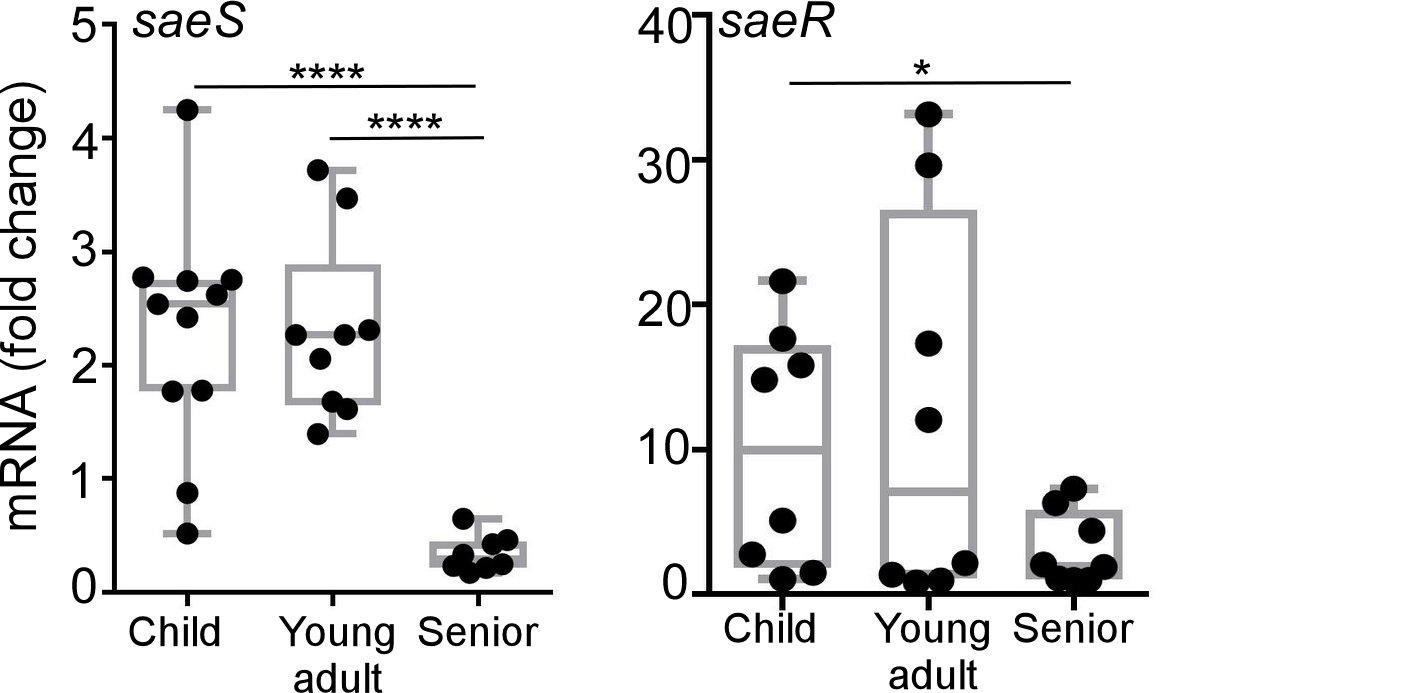
**
